# Chronic administration of AMD3100 increases survival and alleviates pathology in SOD1^G93A^ mice model of ALS

**DOI:** 10.1186/s12974-016-0587-6

**Published:** 2016-05-26

**Authors:** Inna Rabinovich-Nikitin, Assaf Ezra, Beka Barbiro, Polina Rabinovich-Toidman, Beka Solomon

**Affiliations:** Department of Molecular Microbiology and Biotechnology, George S. Wise Faculty of Life Sciences, Tel Aviv University, 69978 Tel Aviv, Israel

**Keywords:** Microglia, Blood-spinal cord barrier, AMD3100, ALS, SOD1^G93A^ mice, Hematopoietic stem and progenitor cells, CXCR4/CXCL12

## Abstract

**Background:**

Amyotrophic lateral sclerosis (ALS) is a progressive fatal neurodegenerative disease, involving both upper and lower motor neurons. The disease is induced by multifactorial pathologies, and as such, it requires a multifaceted therapeutic approach. CXCR4, a chemokine receptor widely expressed in neurons and glial cells and its ligand, CXCL12, also known as stromal-cell-derived factor (SDF1), modulate both neuronal function and apoptosis by glutamate release signaling as well as hematopoietic stem and progenitor cells (HSPCs) migration into the blood and their homing towards injured sites. Inhibition approaches towards the CXCR4/CXCL12 signaling may result in preventing neuronal apoptosis and alter the HSPCs migration and homing. Such inhibition can be achieved by means of treatment with AMD3100, an antagonist of the chemokine receptor CXCR4.

**Methods:**

We chronically treated male and female transgenic mice model of ALS, SOD1^G93A^ mice, with AMD3100. Mice body weight and motor function, evaluated by Rotarod test, were recorded once a week. The most effective treatment regimen was repeated for biochemical and histological analyses in female mice.

**Results:**

We found that chronic administration of AMD3100 to SOD1^G93A^ mice led to significant extension in mice lifespan and improved motor function and weight loss. In addition, the treatment significantly improved microglial pathology and decreased proinflammatory cytokines in spinal cords of treated female mice. Furthermore, AMD3100 treatment decreased blood-spinal cord barrier (BSCB) permeability by increasing tight junction proteins levels and increased the motor neurons count in the lamina X area of the spinal cord, where adult stem cells are formed.

**Conclusions:**

These data, relevant to the corresponding disease mechanism in human ALS, suggest that blocking CXCR4 by the small molecule, AMD3100, may provide a novel candidate for ALS therapy with an increased safety.

**Electronic supplementary material:**

The online version of this article (doi:10.1186/s12974-016-0587-6) contains supplementary material, which is available to authorized users.

## Background

Amyotrophic lateral sclerosis (ALS) is a progressive fatal neurodegenerative disease involving both upper and lower motor neurons [1]. Although the pathology of ALS has not yet been understood, several studies have obtained evidence including increased levels of proinflammatory cytokines and proliferation and activation of glial cells involvement in the disease progression. Microglia cells play a critical role as resident immunocompetent and phagocytic cells within the central nervous system. Microglial activation is associated with increased nitric oxide, reactive oxygen species, and proinflammatory cytokines, such as TNFα and IL-1β, which could generate a neuroinflammatory environment [2, 3]. Recent studies indicate that microglial cells could be involved in the initiation and propagation of motor neuronal cell damage in ALS [4–9]. Indeed, microglial activation was also shown to have a major role in later disease progression, not only in disease onset and early phase of disease progression in mutant SOD1 (mSOD1) mice [10]. Therefore, targeted therapy to microglia may be an effective strategy for ALS treatment.

Recently, the anti-inflammatory effect of bone marrow-derived stem cells (BMSCs) has generated a great deal of interest. Studies have shown that BMSCs can regulate immune cell proliferation, such as shown in animal models of Parkinson disease, cerebral ischemia, Krabbe’s disease, and ALS, in which administration of BMSCs attenuated inflammation and improved motor function [11–15].

Transplantation of BMSCs has been suggested as a potential therapeutic approach to prevent neurodegenerative diseases; however, it remains problematic due to issues of engraftment, potential toxicities, and other different factors [16]. An alternative therapeutical approach is pharmacological-induced recruitment of endogenous BMSCs into an injured tissue by systemic administration of chemokines. Chemokines are a family of small secreted proteins with variety of immune and neural functions, such as regulation of leukocyte trafficking, organization of the hematopoetic/lymphopoetic system, and angiogenesis. Chemokines and their receptors in the central nervous system (CNS) are relevant for the understanding of brain physiology and pathophysiology and may lead to the development of targeted treatments for neurodegenerative diseases [17].

CXCR4 is a chemokine receptor, widely expressed in neurons and glial cell. Its ligand, CXCL12, also known as stromal-cell-derived factor (SDF1), modulates both neuronal function and apoptosis by glutamate release signaling [18] as well as hematopoietic stem and progenitor cells (HSPCs) migration into the blood and their homing in injured sites [19]. Inhibition approaches towards the CXCR4/CXCL12 signaling might result in preventing the toxic cascade of glutamate release from glial cells and neuronal apoptosis and alter the HSPCs migration and homing. Such inhibitor is AMD3100 (Mozobil, plerixafor), a FDA-approved bicyclam molecule that specifically blocks CXCL12 binding to CXCR4. AMD3100 was previously shown to rapidly mobilize hematopoietic stem and progenitor cells (HSPCs) from the bone marrow into the circulating blood for transplantation in patients with hematological malignancies such as non-Hodgkin’s lymphoma or multiple myeloma [19, 20].

Inhibition of CXCR4/CXCL12 signaling may also regulate blood-central nervous system-barrier (BCNSB) integrity through tight junction proteins [21]. In human ALS and rodents expressing SOD1 mutations were reported pathological changes in the BCNSB composed of the blood-brain barrier (BBB), blood-spinal cord barrier (BSCB), and blood-cerebrospinal fluid barrier (BCSFB). All three barriers have an essential role in modulating CNS homeostasis, due to the composition of the microvasculature–capillaries formed by endothelial cells. According to this unique composition, breakdown of the BSCB was proposed to have a primary role in the early-stage disease pathogenesis and its rehabilitation might slow down disease progression [22]. Early studies of serum immunoglobulin (IgG) leakage reported microhemorrhages and BSCB breakdown in the spinal cord of ALS patients and mSOD1 transgenic mice even before motor neurons degeneration, as evident from the mouse model [23–25].

The breakdown of the BSCB in ALS mice is followed by measuring of the levels of various tight junction proteins including ZO-1, occludin, and claudin-5 between endothelial cells [23]. The loss of these tight junction proteins in the microvasculature is mediated by various proinflammatory cytokines such as monocyte chemoattractant protein-1 (MCP1, also known as CCL2), TNF-α, IL-1β, and IFN-γ [23]. SOD1 mutants mediate endothelial damage even before motor neuron death and hypoxia and inflammation lead to increased BSCB permeability and disruption suggesting that early intervention in treatment of the disease may have beneficial effect in rehabilitation of BSCB, in delay of the disease onset and increase of survival [22].

Here, we investigate if chronic administration of AMD3100, a pharmacological antagonist of CXCR4, to transgenic mice model of ALS (SOD1^G93A^) enables to (1) regulate inflammatory response by reducing proinflammatory cytokines and microglial activation, (2) prevent BSCB disruption, and if all together (3) may lead to extension in mice lifespan and improved well-being.

## Methods

### Transgenic mice and treatment

We used transgenic mouse strain expressing mutant human SOD1 with the ALS-causing mutations: hemizygous SOD1^G93A^_,_ which was maintained on a C57BL/6 congenic background [26]. Fifty-, 70-, and 90-day-old female SOD1^G93A^ mice were injected subcutaneously with 5 mg AMD3100 (Sigma-Aldrich, USA) or PBS (Biological Industries, Israel) twice a week. Mice received the treatment till the end stage of the disease, which was defined as the point at which animals could not right themselves within 30 s after being placed on their side. At that point, mice were euthanatized with Co_2_._._ Other groups of 50-day-old SOD1^G93A^ mice and wild type littermate (LM) mice as control were also treated with AMD3100 or PBS and sacrificed at 110 days old via i.p anesthesia administration of 100 mg/kg Ketamine (Fort Dodge, USA) and 20 mg/kg Xylazine (Merck, Germany) following trans-cardial perfusion with saline. The spinal cords that were collected from the sacrificed mice served for biochemical (five mice in each group) and histological analysis (three mice in each group). The animals were in house maintained colony. Mice were housed in standard conditions: constant temperature (22 ± 1 °C), humidity (relative, 40 %), and a 12-h light/dark cycle and were allowed free access to food and water. All of the animal experiments were conducted in accordance with the Guide for the Care and Use of Laboratory Animals and were approved by the Institutional Animal Care and Use Committee of Tel Aviv University.

### Clinical assessment

During the treatment period, mice were weighed and their muscle strength and coordination deficits were evaluated once a week using a Rotarod apparatus (rotating at an increasing speed ranging from 4–40 RPM, with a constant acceleration of 1 RPM per 10 s). Mice were trained on the machine for 3 days before the actual beginning of the analysis. The time each mouse remained on the drum was recorded, up to 300 s in each of the three attempts made. Disease onset was determined as the time animals reached their maximum bodyweight.

### Tissue fractionation

SOD1^G93A^ and LM mice were sacrificed at the age of 110 days, and samples of soluble and membrane enriched fractions were prepared from spinal cord tissues. The tissues were stored at −70 °C until homogenization. Tissues were homogenized on ice in 5 volumes (*w*/*v*) of T-per extraction buffer (Pierce, USA) complemented with protease inhibitor tablets (Complete Mini Protease Inhibitor Tablets, Roche) and phosphatase inhibitor cocktail tablets (phosSTOP, Roche). After sonication, the homogenates were centrifuged at 100,000*g* for 1 h at 4 °C. The resulting supernatants represent the soluble fraction. The pellets were further resuspended in T-per extraction buffer complemented with protease and phosphatase inhibitors as above, 0.5 % triton-100, 1 % sodium deoxycholate, and 3 % SDS. The pellets were sonicated and centrifuged at 10,000*g* for 1 h at 4 °C. Protein concentrations were determined using BCA protein assay kit (Thermo, USA).

### Western immunoblot analysis

Equal amounts of mice spinal cord homogenates protein (40 μg) were resolved separately for soluble and membrane fractions on SDS-PAGE, transferred to nitrocellulose membrane, and blocked overnight with 5 % skim milk in TBS-T (0.3 % Tween 20). Blots of the soluble fraction were probed with the following primary antibodies: mouse anti-actin (1:10,000 Sigma-Aldrich, USA), rabbit anti-MCP-1 (1:1000 Peprotech, USA), rabbit anti-IL-6 (1:1000 Peprotech, USA), and mouse anti-Iba-1 (1:1000 Millipore, Germany). Blots of the membrane fraction were probed with the following primary antibodies: mouse anti-actin (1:10,000 Sigma-Aldrich, USA), rabbit anti-occludin (1:1000 Abcam, UK), rabbit anti-claudin 5 (1:500, Sigma- Aldrich, USA), rabbit anti-ZO-1 (1:1000 Sigma-Aldrich, USA), and rabbit anti-cd36 (1:1000 Abcam, UK). Blots were incubated with corresponding secondary antibodies conjugated peroxidase (Sigma- Aldrich, USA) and developed with the EZ-ECL detection kit (Biological Industries, Israel). Quantitative densitometric analysis was performed using the densitometric software EZQuant-Gel (version 2.12).

### TNF-α measurement

Soluble fraction of spinal cord homogenates, treated with AMD3100 (*n* = 5) or PBS (*n* = 5), was subjected to mouse TNF alpha ELISA Ready-SET-Go! kit, according to the manufacturer’s instructions (eBioscience, USA).

### Evans blue permeability assay

Fifty-day-old female SOD1^G93A^ mice were treated with AMD3100 (*n* = 3) or PBS (*n* = 3). On the age of 110 days, the mice were i.p injected with Evans blue dye (50 μg/g body weight). Three hours post Evans blue injection, the mice were anesthetized i.p with ketamine/xylazine and perfused with saline. Spinal cords were immersed in 500-μl formamide (Sigma- Aldrich, USA) for 48 h at 60 °C to extract the dye, and its presence was measured by OD_620_ [27].

### Histological staining

A separate cohort of SOD1^G93A^ mice treated with AMD3100 (*n* = 3) or PBS (*n* = 3) at 50 days old was sacrificed at 110 days old by i.p anesthesia with ketamine/xylazine and perfusion with saline. Their spinal cords were harvested, fixed in 4 % (*w*/*v*) paraformaldehyde (PFA) in PBS (pH 7.4) and cryoprotected in 30 % sucrose in PBS. Twenty-five-micro meter free-floating cryosections were prepared from lumber spinal cords and stained for Iba-1, hemosiderin deposits, IgG leakage, and thionin for motor neurons in lamina X. For all histological stainings, 15 sections per group were used. After staining, sections were dehydrated in graded alcohol, cleared in xylene and coverslipped with enthelan (Merck, Germany). For Iba-1 staining, sections were dried and coverslipped with mounting media containing DAPI. Images were captured by a CCD color video camera (ProgRes C14, Jenoptic, Jena, Germany) attached to a Leica DMLB microscope (Leica, Germany) and analyzed with Image-J Software (NIH, freeware).

#### Iba-1 staining

Lumbar sections from SOD1^G93A^ mice were blocked with 0.3 % Triton + 0.2 % goat serum for 1 h and incubated over night at 4 °C with 1:500 Iba-1 (Abcam, UK). On the next day, the sections were incubated with anti-rabbit Alexa 546 (Invitrogen, USA) for 2 h, air-dried, and coverslipped with mounting medium with DAPI (Abcam, UK) [28].

#### Prussian blue staining

Lumbar sections from SOD1^G93A^ mice were incubated in a 5 % potassium ferrocyanide and 5 % hydrochloric acid solution (1:1 working solution) for 30 min. The sections were then washed and subsequently counterstained with nuclear fast-red. Hemosiderin shows blue, the nuclei shows red, whereas the cytoplasm shows pink. To access the relative abundance of Prussian blue-positive deposits per section, we divided total numbers of Prussian blue-positive spots by the number of studied sections [23].

#### IgG leakage staining

Serum protein leakage (IgG) was assessed by Vectastain ABC kit (Vector Laboratories) according to the manufacturer’s instructions. Briefly, sections were treated with 3 % H_2_O_2_ in absolute methanol for 25 min, blocked by horse serum for 1 h at room temperature, and incubated with biotynilated secondary antibody for 1 h and avidin HRP for 30 min. The reaction product was visualized using DAB chromogen substrate (Invitrogen, USA).

#### Nissl stain for motor neurons

Nissl bodies were stained with 0.1 % thionin. Results of motor neurons were expressed as the average of total motor neurons counted per section [29].

### Statistical analysis

Survival and disease onset were analyzed using the Kaplan-Meier with log-rank (Mantel-Cox) test. Weight, behavioral analysis, histochemistry, and biochemistry were presented as the mean ± SEM and subjected to unpaired one-tailed Student’s *t* tests. *p < 0.05; **p < 0.01 was considered statistically significant.

## Results

In this study, we examined the ability of the CXCR4 antagonist, AMD3100, to affect microglial pathology and BSCB integrity towards extended lifespan of transgenic mice model of ALS. The SOD1^G93A^ mice model of ALS used in this study expresses gradual upper and lower motor neurons death, leading to impaired motor function and death, representing a close model of disease progression in humans. Female SOD1^G93A^ mice were administered subcutaneously with 5 mg AMD3100 at 50 days old, twice a week. Mice body weight and their motor function, evaluated by Rotarod test, was recorded once a week. A similar therapeutic protocol was applied on another group of SOD1^G93A^ mice, also starting at 50 days old and sacrificed at 110 days old for biochemical and histological analysis of relevant markers.

Survival experiments starting at 50 days old to SOD1^G93A^ mice were performed for both male and female mice, showing the same trend. The biochemical and histological analyses were chosen to be performed only in females rather than males, due to the fact that this model is less aggressive in females [30], making it more plausible to detect AMD3100’s effect on the disease. Moreover, the mechanism of inhibition for AMD3100 is expected to be the same in both genders and is expected to act in the same manner in both genders. Survival plot for male mice is presented in Additional file 1.

### AMD3100 decreases microglial inflammation levels of treated mice

Inflammation in the central nervous system is a common clinical feature in ALS patients and animal models [31]. Activated microglia and its cytotoxic microglial markers, including TNF-α and IL-6 cytokines, Iba-1 and cd36 markers [2, 3, 32, 33], precede astrocyte reactivity [34] and show reduced neuroprotective behavior of mSOD1 microglia [2, 3].

**Fig. 1:**
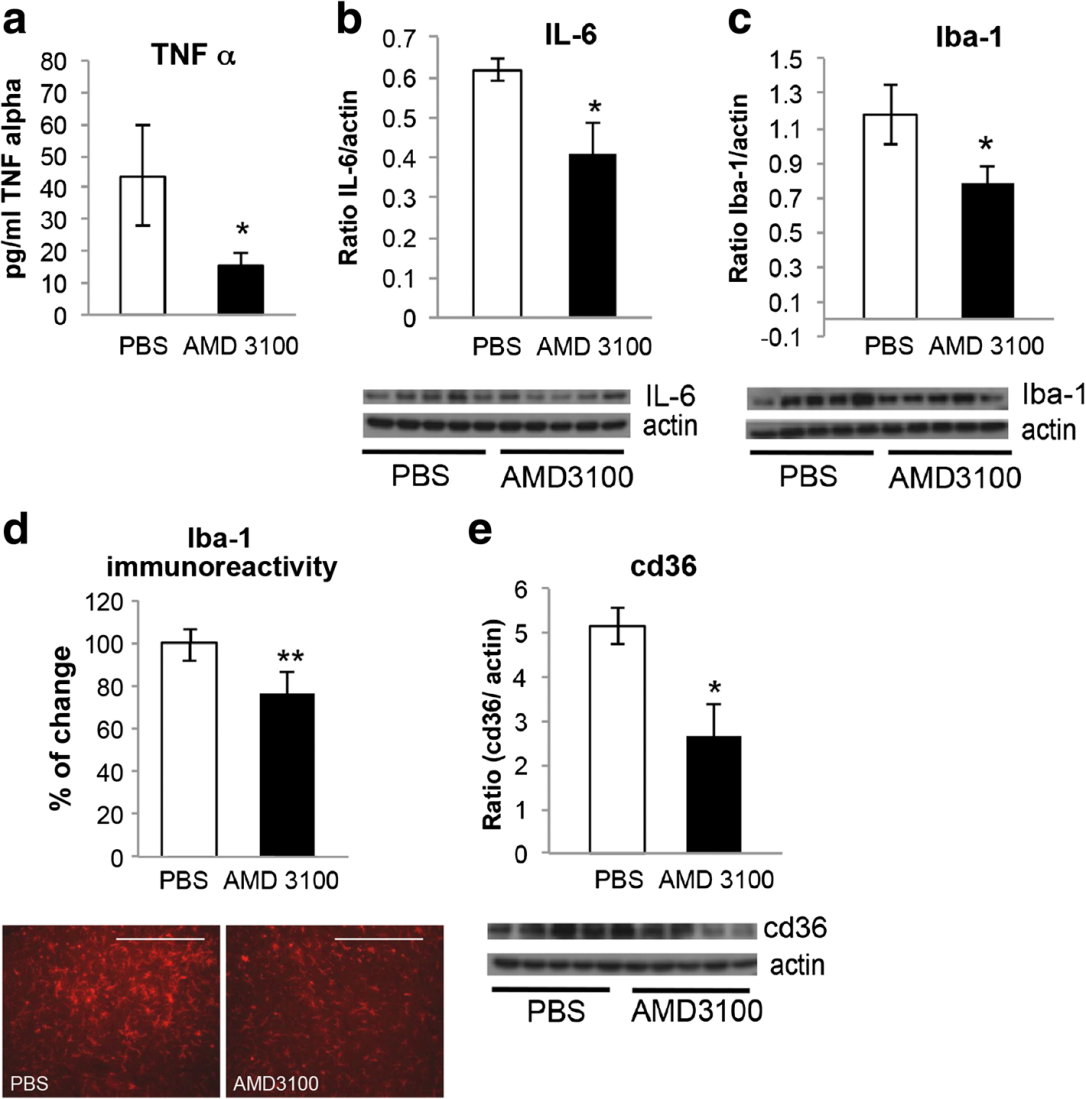
AMD3100 decrease microglial inflammatory markers and proinflammatory markers. SOD1^G93A^ and LM mice were treated with AMD3100 or PBS and sacrificed at 110 days old, and the levels of activated microglia markers were measured. Five mice in each treatment group of SOD1^G93A^ and LM mice were tested. **a** TNF-α level in spinal cord homogenates of SOD1^G93A^ mice were measured using ELISA kit. (TNF-α for PBS is 43.77 pg/ml, for AMD3100 is 15.45 pg/ml) **b** IL-6 levels of SOD1^G93A^ mice measured via western blot analysis. **c** Iba-1 levels of SOD1^G93A^ mice measured via western blot analysis. **d** Immunohistochemical staining of Iba-1. **e** cd36 levels of SOD1^G93A^ mice measured via western blot analysis. Results are mean ± S.E.M., *p < 0.05, **p < 0.01

Indeed, TNF-α levels were significantly reduced in spinal cords of SOD1^G93A^ mice treated with AMD3100, visualized by ELISA kit (Fig. 1a). IL-6 levels were also significantly decreased as visualized by western blot analysis (Fig. 1b), however, in a lower extent than TNF-α. The activated microglial markers Iba-1 (Fig. 1c) and cd36 (Fig. 1e) were significantly decreased as well in treated SOD1^G93A^ mice, visualized by western blot analyses. Immunhistochemical staining of Iba-1 showed the same trend as the immunoblot of this marker (Fig. 1d).

### AMD3100 treatment decreases BSCB permeability and increases tight junction proteins levels

**Fig. 2:**
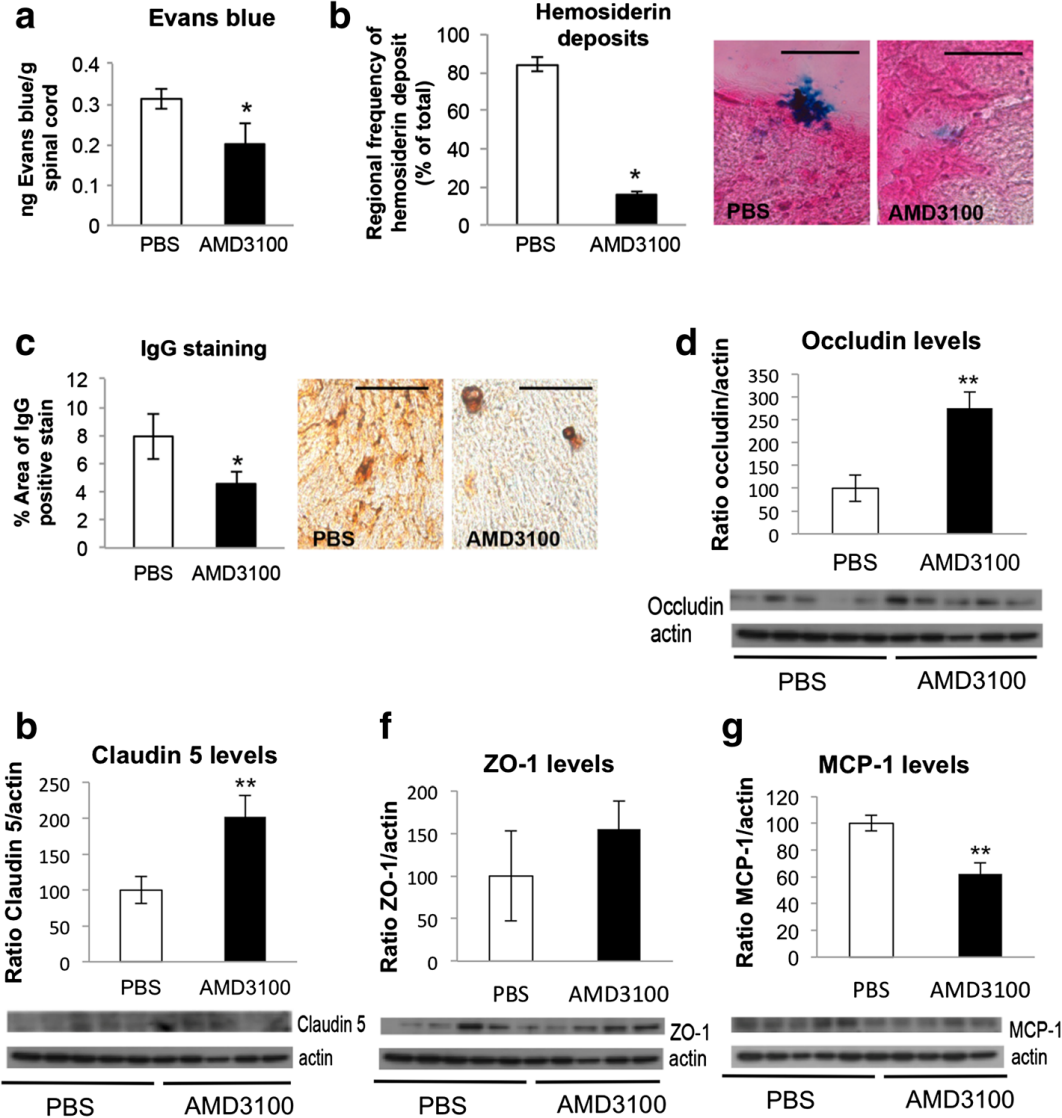
AMD3100 reduces BSCB permeability and increases tight junction proteins levels. **a** Evans blue permeability assay. Fifty-day-old SOD1^G93A^ mice were treated with AMD3100 (*n* = 3) or PBS (*n* = 3). One hundred ten-day-old mice were IP injected with Evans blue dye (50 ug/g body weight). Three hours post injection mice were sacrificed and perfused, and dye presence in the spinal cord was measured as ratio of tissue weight. **b** Regional analysis of hemosiderin deposits. **c** IgG leakage in the lumbar cord anterior horn of SOD1^G93A^ mice treated with AMD3100 (*n* = 3) or PBS (*n* = 3) from 50 to 110 days old. Fifteen nonadjacent sections were examined for each treatment group. **d** Occludin levels of SOD1^G93A^ mice. **e** Claudin-5 levels of SOD1^G93A^ mice. **f** ZO-1 levels of SOD1^G93A^ mice. **g** MCP-1 levels of SOD1^G93A^ mice. For tight junction proteins, SOD1^G93A^ mice were treated with AMD3100 or PBS and sacrificed at 110 days old, and protein levels were measured using western blot. Five mice in each treatment group of SOD1^G93A^ mice were tested. Results are mean ± S.E.M., *p < 0.05, **p < 0.01

Evaluation of BSCB permeability was performed by Evans blue permeability assay and histological staining of hemosiderin deposits and IgG localization. SOD1^G93A^ mice treated with AMD3100 showed significantly less positive Evans blue dye in their spinal cords (Fig. 2a), compared with mice treated with PBS, as well as reduced hemosiderin deposits (Fig. 2b) and reduced IgG leakage levels (Fig. 2c).

ALS-linked SOD1 mutant mice are known to reduce the levels of three major tight junction proteins between endothelial cells, including occludin, claudin-5, and ZO-1, thus disrupting the BSCB integrity [23]. Treatment with AMD3100 increased the levels of all three markers in SOD1^G93A^ (Fig. 2d–f).

MCP-1 is involved in recruiting monocytes and macrophages to the CNS [35]. However, a growing body of evidence implicates MCP-1 as an angiogenic factor as well, being responsible for brain permeability, by affecting the permeability of endothelial cells [36]. Western blot analysis of SOD1^G93A^ mice treated with AMD3100 showed significant reduction in MCP-1 levels, compared with PBS-treated mice (Fig. 2g).

### AMD3100 increases number of motor neurons in the ependymal layer within lamina X of the spinal cord

**Fig. 3:**
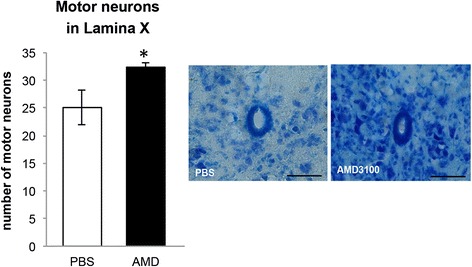
Increase in the number of motor neurons in lamina X following AMD3100 treatment. SOD1^G93A^ mice were treated with AMD3100 (*n* = 3) or PBS (*n* = 3) starting at 50 days old and sacrificed at 110 days old. Fifteen nonadjacent sections per group of lumbar spinal cords were stained with thionin and analyzed within the lamina X zone of central canal. Results are mean ± S.E.M., *p < 0.05

Adult stem cells in the spinal cord appear in the ependymal layer within lamina X around the central canal [37]. Administration of AMD3100 to SOD1^G93A^ mice starting at 50 days old, significantly increased the number of motor neurons in the lamina X, as observed by thionin staining (Fig. 3).

### AMD3100 extends significantly SOD1^G93A^ mice lifespan and disease onset and improves significantly the weight loss and motor function

**Fig. 4:**
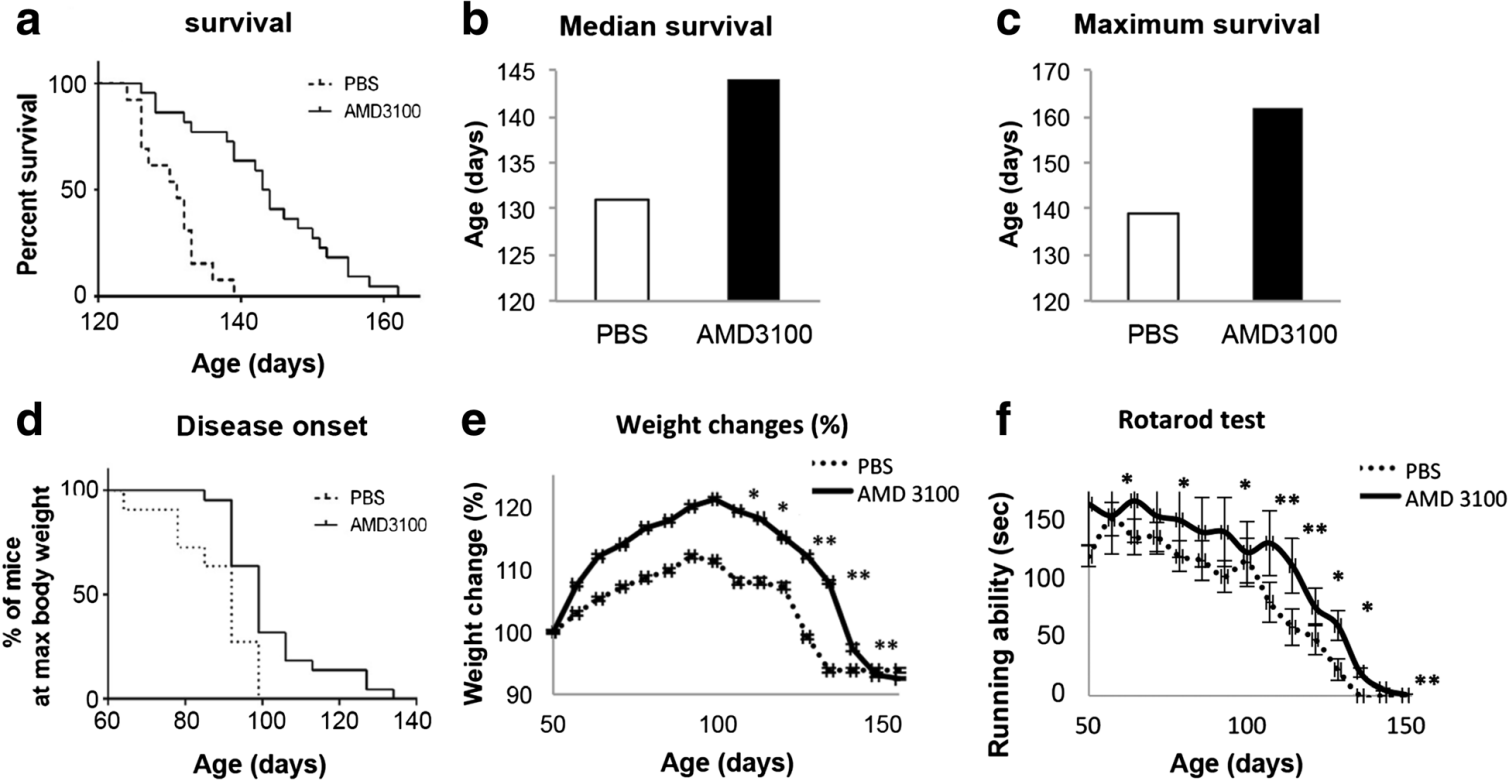
AMD3100 administration significantly extended 50 days old SOD1^G93A^ mice survival and delayed the disease onset. **a** Survival of mice treated with AMD3100 (*n* = 22) and PBS (*n* = 13) was defined as the point at which animals could not right themselves within 30 s after being placed on their side. Mantel-Cox test; *p* < 0.0001. **b** Median survival of mice demonstrates 13 days increase in lifespan. **c** Maximum survival of mice. **d** Disease onset was determined as the time animals reached their maximum bodyweight. Mantel-Cox test; *p* = 0.0036 **e** Weight change monitored weekly. **f** Motor function assessed weekly by Rotarod test. Results are mean ± S.E.M., *p < 0.05, **p < 0.01

Starting at 50 days old, female SOD1^G93A^ mice were treated subcutaneously with 5 mg AMD3100 or PBS twice a week. Mice received continuous treatment until the end stage of disease. Treatment with AMD3100 significantly extended the survival of SOD1^G93A^ mice. Median survival of PBS-treated SOD1^G93A^ mice was 131 days (*n* = 13), whereas treatment with AMD3100 increased the lifespan of SOD1^G93A^ mice to 144 days (*n* = 22) (*p* < 0.0001, Mantel-Cox test), an increase of 13 days (Fig. 4a). Furthermore, treatment with AMD3100 significantly delayed disease onset (Fig. 4d), determined as the time animals reached their maximum bodyweight. Disease progression and mice well-being were monitored weekly by weight record and motor performance by Rotarod. AMD3100 treatment resulted in significant slowdown in weight loss (Fig. 4e) and significant improvement in Rotarod performance (Fig. 4f).

### AMD3100 treatment extends survival of 70 days old mice, but not the onset, whereas treatment at 90 days old has no beneficial effect

**Fig. 5:**
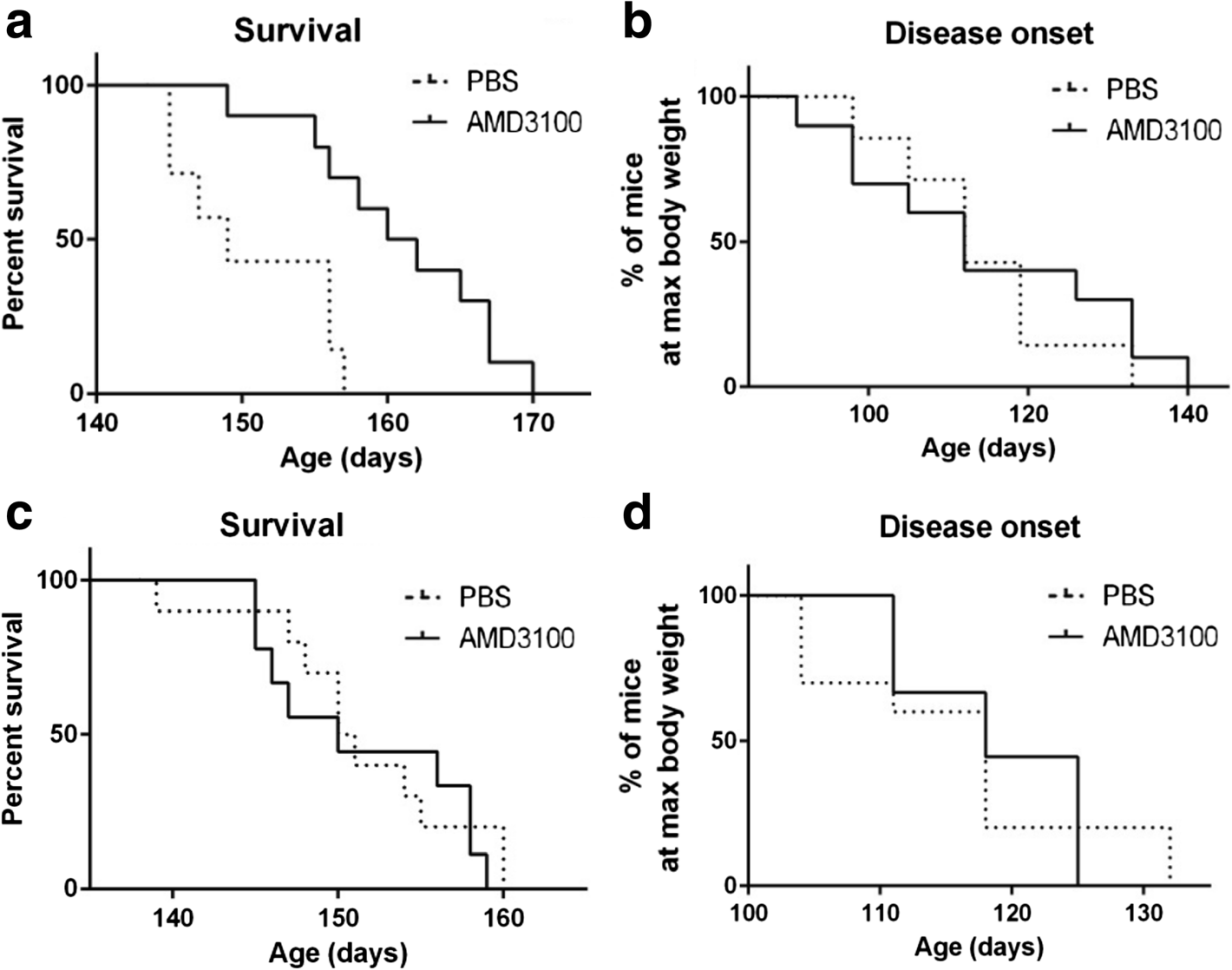
AMD3100 administration had little effect on 70- and 90-day-old SOD1^G93A^ mice survival and disease onset. **a** Survival of mice treated with AMD3100 (*n* = 10) and PBS (*n* = 7) at 70 days old. Mantel-Cox test; *p* = 0.0025. **b** Disease onset of mice treated at 70 days old. **c** Survival of mice treated with AMD3100 (*n* = 9) and PBS (*n* = 10) at 90 days old. **d** Disease onset of mice treated at 90 days old

In order to assess the effect of AMD3100 on later stages of the disease, 70- and 90-day-old female SOD1^G93A^ mice were treated subcutaneously with 5 mg AMD3100 or PBS twice a week. Treatment at 70 days old significantly increased lifespan of mice by 12 days (*p* = 0.0025, Mantel-Cox test). Median survival of PBS-treated SOD1^G93A^ mice was 149 days (*n* = 7), whereas treatment with AMD3100 increased the lifespan of SOD1^G93A^ mice to 161 days (*n* = 10) (Fig. 5a). No significant effect on disease onset (Fig. 5b) was observed. Whereas treatment at 50 and 70 days old increased SOD1^G93A^ mice lifespan, treatment at the late stage of disease, 90 days old, had no effect on survival (Fig. 5c) and disease onset (Fig. 5d). Since treatments at 70 and 90 days old had no significant effect on weight changes and motor function, the biochemical and histological analyses were performed only on female SOD1^G93A^ mice treated with AMD3100 at 50 days old.

## Discussion

Inflammation abnormalities are considered to be highly involved in the neuropathology of ALS. At sites of motor neuron injury, neuroinflammation is a prominent pathological finding characterized by microglial activation, astrogliosis, and infiltration of monocytes and T cells [2, 3]. A study by Hensley et al. suggested that TNF-α, IL6, and IFN cytokines synergize to produce disproportionate microglial activation in the degenerating spinal cord. Moreover, TNF-α alone is a potent stimulant for generating reactive nitrogen species (RNS) and its effects are strongly amplified by the presence of IL-6 or IFN [38]. Evidence for TNF-α involvement in ALS is also supported by high levels of circulating TNF-α and its soluble receptors in ALS patients [39]. This elevation in TNF-α levels may be facilitated by the activation of the CXCR4 pathway on glial cells. Binding of CXCL12 to CXCR4 leads to the downstream release of TNF-α from cell surface. TNF-α then binds to its receptor, TNFR1, on the glial membrane, and in turn triggers glutamate release that eventually leads to neuronal death [40]. Following the downstream activation of the CXCR4 pathway, it can be assumed that inhibition of CXCR4 by AMD3100 may reduce TNF-α levels. Indeed, we demonstrated that chronic administration of AMD3100 to SOD1^G93A^ mice led to a significant decrease in TNF-α levels in the soluble fraction of spinal cords.

IL-6 is also increased in CSF, serum, and skin of ALS patients [41, 42]. The transcriptional regulation of IL-6 is mediated by phosphorylation of ERKs and binding of nuclear factor-kappa B (NF-kB) to the IL-6 promoter. This pathway is enhanced by CXCL12 [43]. Recently, it was reported that treatment with AMD3100 after experimental stroke reduce IL-6 levels as well as other proinflammatory cytokines [44]. In agreement with these findings, our results show that chronic treatment with AMD3100 significantly reduced IL-6 levels in SOD1^G93A^ mice.

Microglial activation was suggested to precede astrocyte reactivity and correlates with disease progression [34]. Activation of microglia may be observed through the up-regulation of Iba1 and cd36 markers [3, 32]. AMD3100 treatment, starting at 50 days, significantly attenuated microglial activation of SOD1^G93A^ mice, observed by reduced protein levels of Iba1 and cd36 in both western blot and immunohistochemical staining. The fact that no effect on the microglial markers was observed in LM mice suggests that this effect is specific to neuropathological processes of disease progression (Additional file 2). Astrocytic markers including GFAP and S100B were not changed in SOD1^G93A^ mice following AMD3100 treatment (Additional file 3).

Accumulating reports state that increased permeability of the BSCB is also involved in motor neuron degeneration and the pathogenesis of ALS. Recent works have strongly confirmed this observation in both human ALS patients [45–47] and SOD1 mutant animals [23, 25, 48]. Here, we show that AMD3100 treatment to SOD1^G93A^ mice led to a significant increase in the expression of tight junction proteins occludin and claudin-5. These findings are further supported by reduction in Evans blue extravasation, decreased hemosiderin deposits, and IgG leakage into the spinal cord. Combined, these results indicate that AMD3100 treatment attenuates the breakdown of BSCB integrity, associated with ALS pathology, and reduces its permeability.

The effect on BSCB integrity is also accompanied by a significant reduction in MCP-1 levels in treated spinal cords. Since CCR2, the receptor for MCP-1, is expressed by endothelial cells, it has been suggested that MCP-1, on top of being an inflammatory marker, has additional functions related to endothelial cells, such as redistributing the tight junction proteins occludin, claudin-5, and ZO-1 [36]. Indeed, Zhong et al. showed that MCP-1 levels are increased at symptomatic and end stage of disease, when the BSCB is already disrupted in animal mutant SOD1 mice.

The three tight junction proteins, occludin, claudin-5 and ZO-1, as well as MCP-1 levels were evaluated in LM-treated mice as well (Additional file 4). This effect of AMD3100 on BSCB permeability of LM might have potential therapeutic effect in other diseases involving BSCB breakdown.

To further stress the multifaceted mechanism of AMD3100, the observed reduction in microglial markers by AMD3100 treatment may have resulted from (1) attenuated infiltration of peripheral cells into the CNS, as BSCB permeability was repaired, (2) reduced infiltration of monocytes/macrophages productions independent of microglia, and (3) AMD3100 suppressed both microglia and macrophage (and/or other infiltrating cell) inflammatory cytokine production.

Spinal cord disorders are characterized by an increase in proliferation of neural progenitor cells (NPC) but preferentially differentiate into glial cells rather than neurons [49]. Thus, in order to enhance NPC proliferation towards neurons, further stimuli should be introduced. A recent study provided such stimulus by lithium administration to SOD1^G93A^ mice. It showed an increase in motor neurons count in the lamina X region, which possesses a stem cell-like activity [29]. In the current study, we demonstrated that AMD3100 treatment resulted in significant increase in motor neurons counts in the lamina X area. Moreover, increase in the number of motor neurons in all area of the spinal cord sections was also observed but was not statistically significant (Additional file 5).

To summarize, three different groups of mice were treated with 5 mg AMD3100, starting at 50, 70, and 90 days old. The best improvement in all parameters, including the clinical signs (weight loss and motor function) was observed at the earliest point of treatment, 50 days old. Treatment at 70 days old followed the same survival rate as 50 days old mice; however, it did not show the same improvement in clinical signs. Starting treatment at 90 days old mice had no clinical benefits whatsoever. These results stress the importance of early detection in ALS patients for AMD3100 treatment to be effective. For maximal benefits from AMD3100 treatment, appropriate diagnosis of ALS at early stages must be developed.

## Conclusions

Here, we show that chronic administration of AMD3100 to transgenic mice model of ALS (SOD1^G93A^) has a substantial clinical feature via restoration of levels of tight junction proteins that are essential for BSCB integrity and reduction of inflammation, leading to significant increase in survival of treated mice. The data presented here, relevant to the corresponding disease mechanism in humans, implicates AMD3100 as a possible candidate for ALS therapy with a multifaceted effect. As soon as a full understanding of its effects is reached, treatment based on this agent can move forward relatively fast in the pipeline, as AMD3100 was found safe and approved by the FDA for other indications.

## Additional files

Additional file 1: AMD3100 administration significantly extended 50 days old SOD1^G93A^ mice survival males. Survival of mice treated with AMD3100 (*n*=12) and PBS (*n*=12) was defined as the point at which animals could not right themselves within 30 sec after being placed on their side. Mantel-Cox test; *p* < 0.0001. (TIF 91 kb) Additional file 2: AMD3100 does not affect microglial inflammatory markers and proinflammatory markers in LM. LM mice were treated with AMD3100 or PBS, sacrificed at 110 days old, and levels of activated microglia markers were measured. Five mice in each treatment group of SOD1^G93A^ and LM mice were tested. **a**. TNF-α level in spinal cord homogenates of SOD1^G93A^ mice were measured using ELISA kit. **b**. IL-6 levels of SOD1^G93A^ mice measured via western blot analysis. **c**. Iba-1 levels of SOD1^G93A^ mice measured via western blot analysis. **d**. cd36 levels of SOD1^G93A^ mice measured via western blot analysis. Results are mean ± S.E.M. (TIF 87 kb) Additional file 3: Astrocytic markers including GFAP and S100B were not changed in SOD1^G93A^ mice following AMD3100 treatment. SOD1^G93A^ mice were treated with AMD3100 or PBS, sacrificed at 110 days old, and levels of astrocytes markers were measured. Five mice in each treatment group of SOD1^G93A^ and PBS were tested. **a**. GFAP levels of SOD1^G93A^ mice measured via western blot analysis. **b**. S100B levels of SOD1^G93A^ mice measured via western blot analysis. Results are mean ± S.E.M. (TIF 38 kb) Additional file 4: AMD3100 increases tight junction proteins levels in LM. For tight junction proteins LM mice were treated with AMD3100 or PBS, sacrificed at 110 days old, and protein levels were measured using western blot. Five mice in each treatment group of SOD1^G93A^ mice were tested. **a**. occludin levels of LM mice. **b**. claudin-5 levels of LM mice. **c**. ZO-1 levels of SOD1^G93A^ mice. **d**. MCP-1 levels of SOD1^G93A^ mice. Results are mean ± S.E.M, **p*  < 0.05. (TIF 85 kb) Additional file 5: Increase in the number of motor neurons in spinal cords of AMD3100 following treatment. SOD1^G93A^ mice were treated with AMD3100 (*n*=3)or PBS (*n*=3) starting at 50 days old and sacrificed at 110 days old. Fifteen nonadjacent sections per group of lumbar spinal cords were stained with thionin and analyzed. Results are mean ± S.E.M. (TIF 191 kb)

## Abbreviations

ALS: amyotrophic lateral sclerosis
BBB: blood-brain barrier
BCNSB: blood-central nervous system barrier
BCSFB: blood-cerebrospinal fluid barrier
BMSCs: bone marrow-derived stem cells
BSCB: blood-spinal cord barrier
CNS: central nervous system
HSPCs: hematopoietic stem and progenitor cells
IgG: immunoglobulin
LM: littermate
MCP1: monocyte chemoattractant protein-1
mSOD1: mutant SOD1
NF-kB: nuclear factor-kappa B
NPC: neural progenitor cells
PFA: paraformaldehyde
RNS: reactive nitrogen species
SDF1: stromal-cell-derived factor
