# Supplementary figures and images for: Chronic administration of AMD3100 increases survival and alleviates pathology in SOD1G93A mice model of ALS
Source: J Neuroinflammation. 2016 May 26;13:123. doi: 10.1186/s12974-016-0587-6 (PMC4882847; doi:10.1186/s12974-016-0587-6)

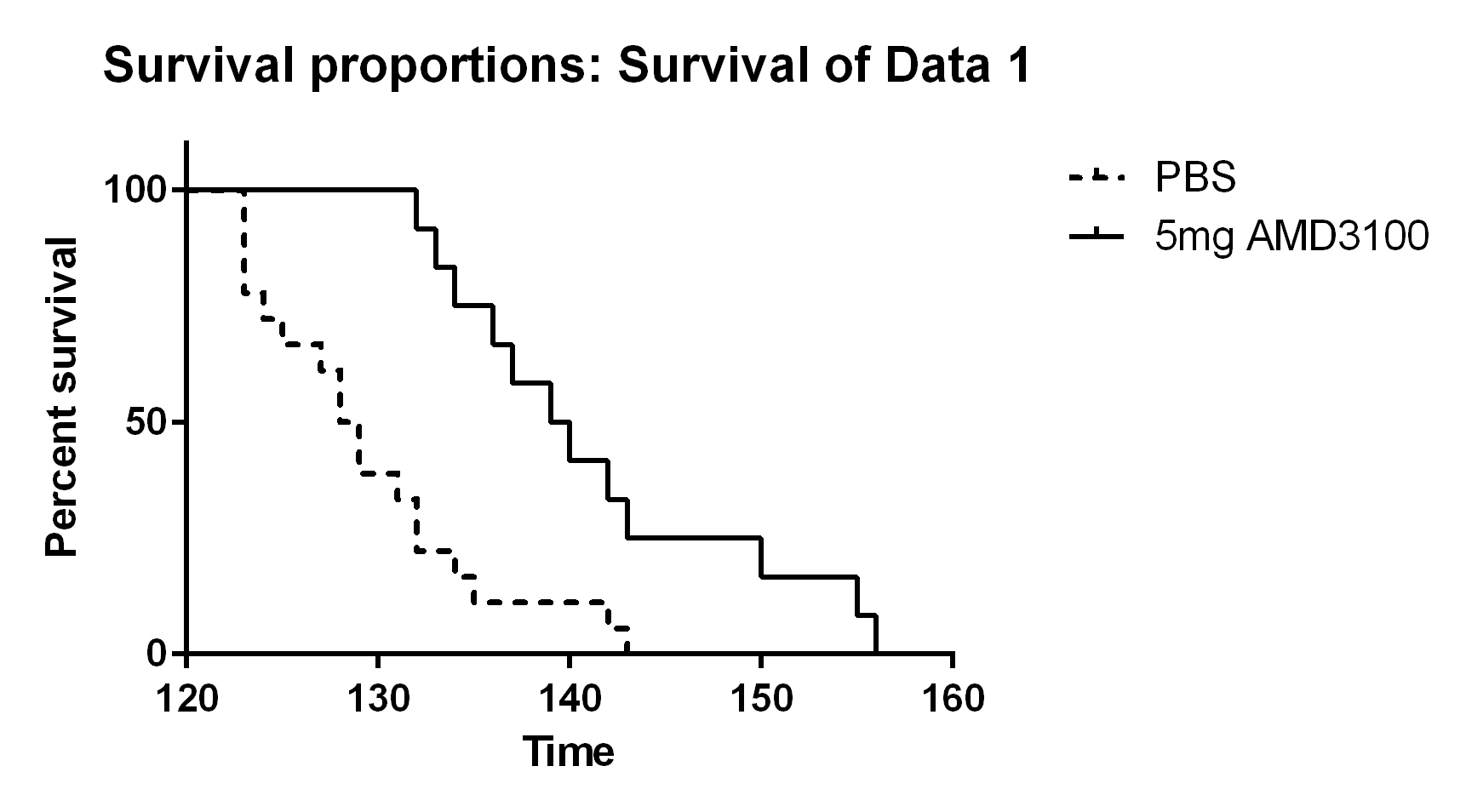

Supplement: Additional file 1: — AMD3100 administration significantly extended 50 days old SOD1G93A mice survival males. Survival of mice treated with AMD3100 (n=12) and PBS (n=12) was defined as the point at which animals could not right themselves within 30 sec after being placed on their side. Mantel-Cox test; p < 0.0001. (TIF 91 kb) [file 12974_2016_587_MOESM1_ESM.tif]

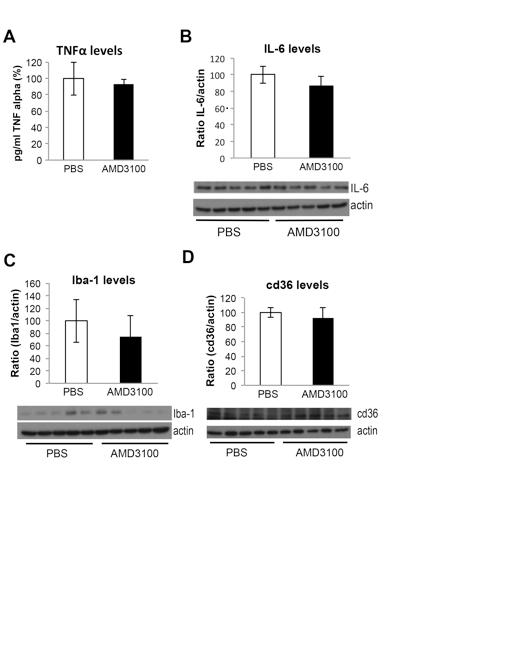

Supplement: Additional file 2: — AMD3100 does not affect microglial inflammatory markers and proinflammatory markers in LM. LM mice were treated with AMD3100 or PBS, sacrificed at 110 days old, and levels of activated microglia markers were measured. Five mice in each treatment group of SOD1G93A and LM mice were tested. a. TNF-α level in spinal cord homogenates of SOD1G93A mice were measured using ELISA kit. b. IL-6 levels of SOD1G93A mice measured via western blot analysis. c. Iba-1 levels of SOD1G93A mice measured via western blot analysis. d. cd36 levels of SOD1G93A mice measured via western blot analysis. Results are mean ± S.E.M. (TIF 87 kb) [file 12974_2016_587_MOESM2_ESM.tif]

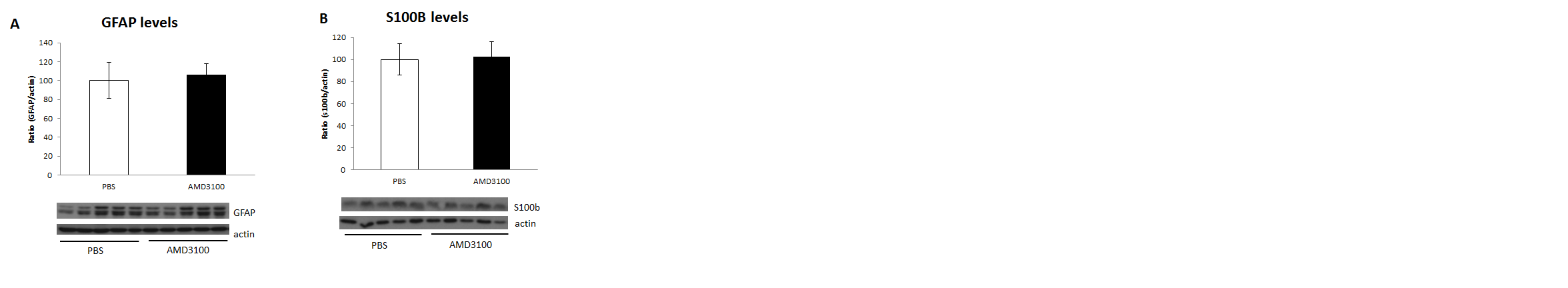

Supplement: Additional file 3: — Astrocytic markers including GFAP and S100B were not changed in SOD1G93A mice following AMD3100 treatment. SOD1G93A mice were treated with AMD3100 or PBS, sacrificed at 110 days old, and levels of astrocytes markers were measured. Five mice in each treatment group of SOD1G93A and PBS were tested. a. GFAP levels of SOD1G93A mice measured via western blot analysis. b. S100B levels of SOD1G93A mice measured via western blot analysis. Results are mean ± S.E.M. (TIF 38 kb) [file 12974_2016_587_MOESM3_ESM.tif]

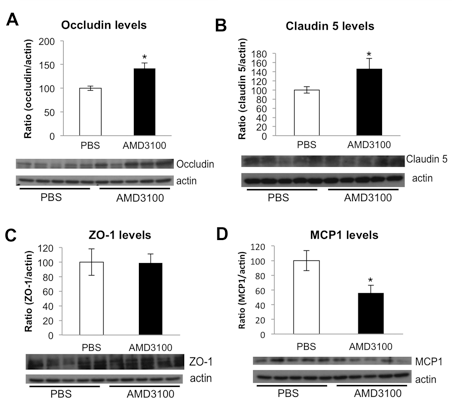

Supplement: Additional file 4: — AMD3100 increases tight junction proteins levels in LM. For tight junction proteins LM mice were treated with AMD3100 or PBS, sacrificed at 110 days old, and protein levels were measured using western blot. Five mice in each treatment group of SOD1G93A mice were tested. a. occludin levels of LM mice. b. claudin-5 levels of LM mice. c. ZO-1 levels of SOD1G93A mice. d. MCP-1 levels of SOD1G93A mice. Results are mean ± S.E.M, *p < 0.05. (TIF 85 kb) [file 12974_2016_587_MOESM4_ESM.tif]

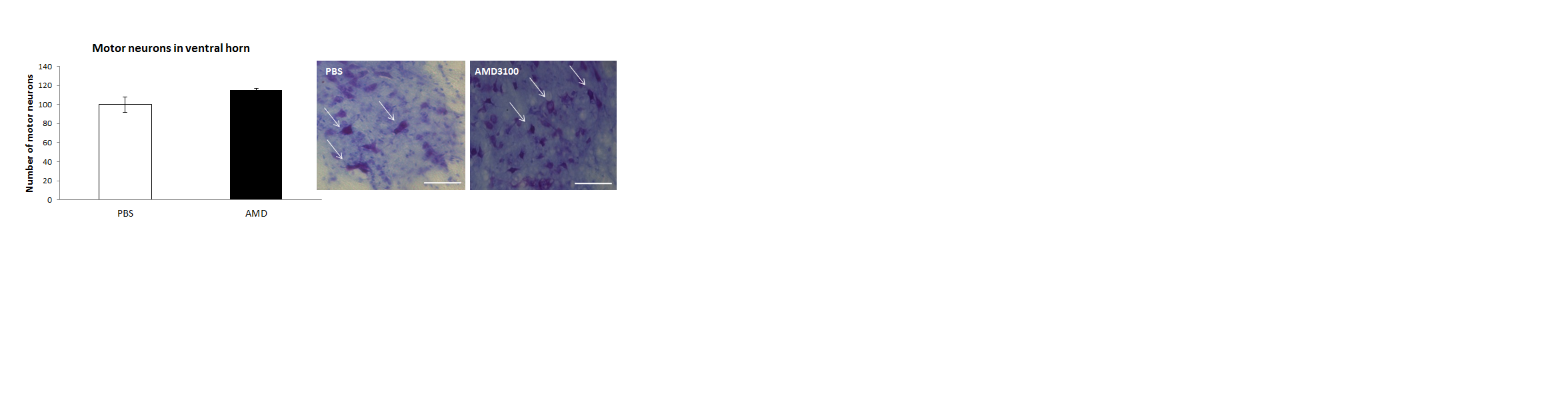

Supplement: Additional file 5: — Increase in the number of motor neurons in spinal cords of AMD3100 following treatment. SOD1G93A mice were treated with AMD3100 (n=3)or PBS (n=3) starting at 50 days old and sacrificed at 110 days old. Fifteen nonadjacent sections per group of lumbar spinal cords were stained with thionin and analyzed. Results are mean ± S.E.M. (TIF 191 kb) [file 12974_2016_587_MOESM5_ESM.tif]
